# Supplementary material for: Brain-age models with lower age prediction accuracy have higher sensitivity for disease detection
Source: PLoS Biol. 2025 Oct 28;23(10):e3003451. doi: 10.1371/journal.pbio.3003451 (PMC12633945; doi:10.1371/journal.pbio.3003451)
Supplement: S1 Text — (PDF) [file pbio.3003451.s001.pdf]

Brain-age models with lower age prediction accuracy have higher sensitivity for disease detection

Supplementary Online Material

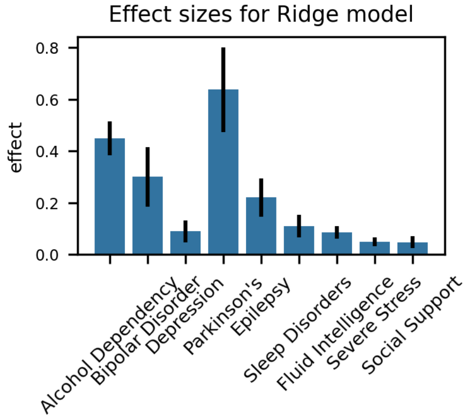

Figure A: Effect Sizes for Various Conditions Using the Ridge Regression Model

This bar plot displays the effect sizes for different neurological conditions, psychiatric disorders, and cognitive/environmental factors when using the ridge regression model for brain age prediction. The x-axis lists the conditions, including alcohol dependency, bipolar disorder, depression, Parkinson's disease, epilepsy, sleep disorders, fluid intelligence, severe stress, and social support. The y-axis represents the effect size, indicating the magnitude of the difference in brain age gap between individuals with the condition and matched controls.

Error bars represent the standard error of the mean (SEM) derived from bootstrapping over participants. However, it's important to note that this uncertainty measure is not the primary focus of our analyses. These error bars indicate how the effect size might generalize to new cohorts, whereas our main interest lies in how the effect size would generalize to new models applied to the same cohort. For our core analyses, we employ a different approach to assess uncertainty across models rather than across participants.

Larger effect sizes suggest a greater impact of the condition on brain aging as measured by the model.

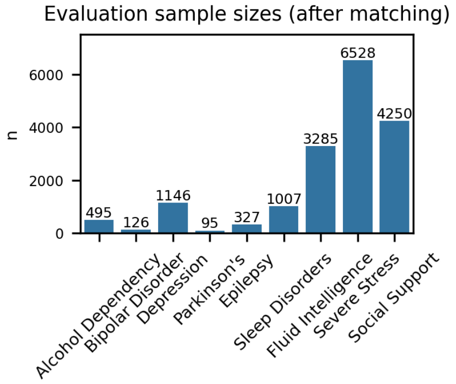

Figure B: Evaluation Sample Sizes After Propensity Score Matching

The sample sizes represent the number of participants retained after propensity score matching, where participants were matched based on relevant demographic and clinical variables. Participants were excluded if no quality match was found within the specified caliper of the propensity score matching procedure. These sample sizes reflect the available data for each condition after the matching process.

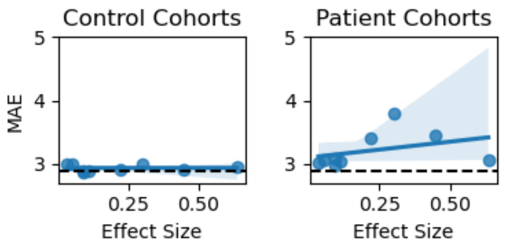

Figure C: Comparison of Effect Size vs.
Mean Absolute Error (MAE) in Control and Patient Cohorts

This figure compares the relationship between effect size and Mean Absolute Error (MAE) in the matched control cohorts (left panel) and patient cohorts (right panel) for the ridge regression model. Each point represents a specific disease or condition. The dashed line indicates the model's overall MAE on the full healthy test set. In the patient cohort plot, the solid line represents the linear trend of MAE as effect size increases, with the shaded area showing the 95% confidence interval. This comparison highlights the differing patterns of prediction errors between healthy controls and patients across various effect sizes.

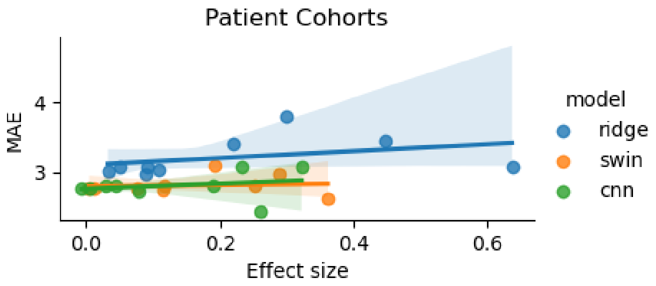

Figure D: Relationship between Effect Size and
Mean Absolute Error (MAE) across Different Models

This figure illustrates the relationship between effect size and Mean Absolute Error (MAE) for three different brain age prediction models (ridge regression, SWIN transformer, and CNN) in patient cohorts. Each point represents a specific disease or condition. The x-axis shows the effect size, indicating the magnitude of the brain age gap between patients and matched controls. The y-axis shows the MAE of age prediction for each model on the respective patient cohort. The lines represent the linear trend for each model, with shaded areas indicating the 95% confidence interval. The plot demonstrates how prediction errors vary with effect size across different model complexities. Notably, the ridge regression model shows a stronger positive relationship between effect size and MAE compared to the more complex SWIN and CNN models, suggesting that simpler models may be more sensitive to disease-related brain changes.

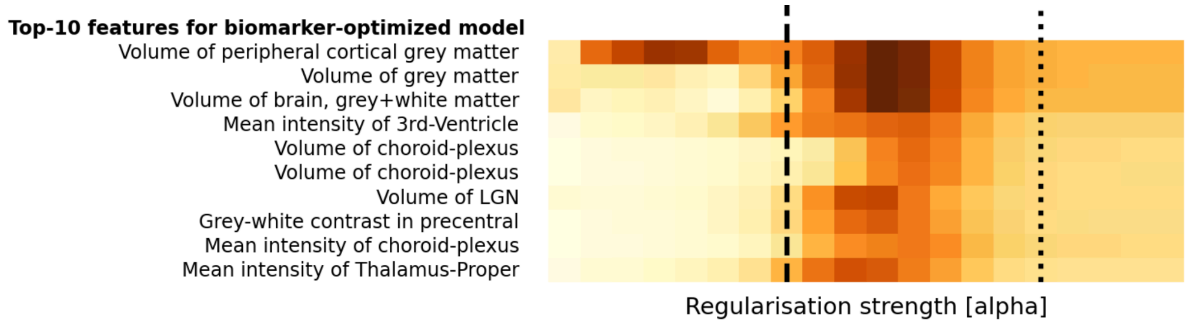

Supplementary Figure E: Feature importances for stronger regularization

Main text Figure 4 shows the top-10 features for a regularization strength maximizing brain-age-gap effect sizes for the majority for conditions. Here, we show an even higher level of regularization for comparison. The trend towards global features holds, with only minor differences in the top-10 features.

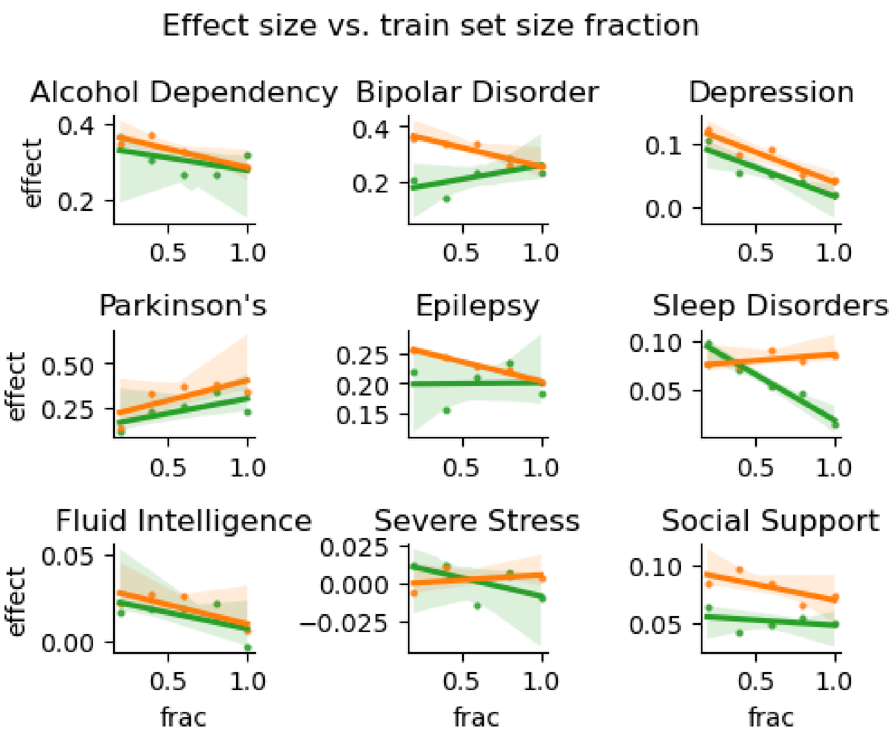

Supplementary Figure F, Panel A

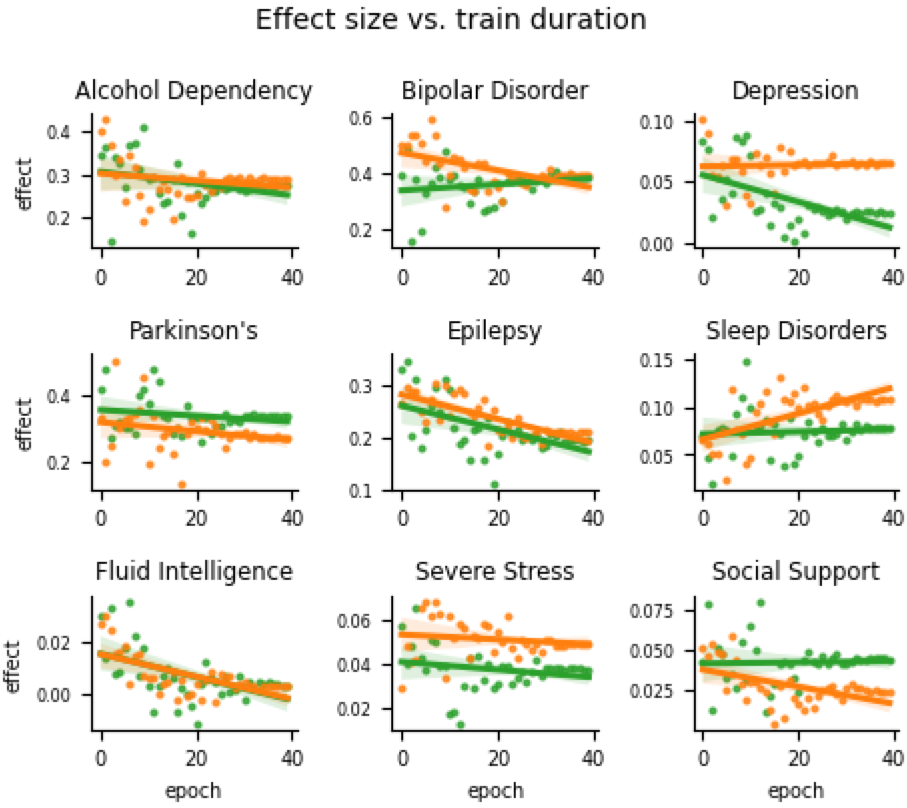

Supplementary Figure F, Panel B

Supplementary Figure F: Training set size and training duration effects across conditions Effect sizes for nine target conditions comparing Swin Transformer (orange) and CNN (green) architectures under varying training conditions. Panel 6a shows effect sizes as a function of training set fraction (0.3 to 1.0 of full training data). Panel 6b shows effect sizes across training epochs (0-40). Points represent individual

measurements, lines show linear trends with 95% confidence intervals derived from bootstrap resampling.

The predominant pattern across most conditions is a decline in effect sizes with increased training data or duration, particularly evident in Depression, Epilepsy, and Alcohol Dependency. This supports our main finding that models optimized for age prediction often become less sensitive to disease-related brain patterns. While some conditions appear to show different patterns (e.g., Parkinson's disease and Bipolar Disorder in training set size analysis), closer inspection reveals these apparent exceptions are primarily driven by high variability in small sample conditions, resulting in less reliable trend estimates. The high variance in these cases, visible in the wider confidence intervals and scattered individual measurements, suggests these divergent patterns should be interpreted with caution.

Sleep Disorders presents a more robust exception, with the Swin Transformer maintaining or increasing effect sizes with more training. Non-clinical targets (Fluid Intelligence, Severe Stress, Social Support) generally show smaller effect sizes and less pronounced training effects, possibly reflecting more subtle brain structural variations compared to clinical conditions. The consistency of declining effect sizes across most conditions and both model architectures, when considering only reliable estimates, reinforces our core finding about the trade-off between age prediction accuracy and disease detection sensitivity.

Supplementary Note A: On Methodological Choices

1. On the Use of Whole-Dataset Standardization

A query was raised regarding our use of whole-dataset feature standardization, noting that standard machine learning practice often involves fitting a scaler on the training set and applying it to test data to prevent information leakage. While this is a valid concern in many predictive modeling contexts, our approach is methodologically justified by the specific nature of our data and analytical goals.

Common Practice in Large-Scale Neuroimaging Research

In large-scale neuroimaging research, whole-dataset scaling is not only common but often standard practice. Smith et al. (2018) applied standardization across the entire dataset when developing imaging-derived phenotypes for the UK Biobank, and Alfaro-Almagro et al. (2018) similarly describe normalization procedures applied to the full dataset. Elliott et al. (2018) and Cole et al. (2018) have also used whole-dataset scaling in influential neuroimaging studies.

This practice acknowledges a fundamental characteristic of large-scale neuroimaging data: the remarkable stability of feature distributions. With 46,000 participants - and our training set comprising 27,000 of those (approximately 59%) - the means and standard deviations calculated from the training set alone are virtually identical to those derived from the full dataset. In such massive cohorts, imaging features effectively function as population constants rather than variables that differ significantly between samples.

Any Potential Bias Works Against Our Central Finding

The most compelling counterargument is that any bias introduced by whole-dataset scaling would work *against* our primary finding. Our central discovery is that models with lower age prediction accuracy tend to yield better disease detection. If whole-dataset scaling provided an advantage, it would serve only to slightly improve the linear models' age prediction accuracy. This hypothetical boost would further accentuate the inverse relationship that we observe - that is, if our linear models were even more accurate, then the paradoxical gap between accuracy and disease detection sensitivity would be even larger. In other words, our results represent a conservative estimate; any potential bias would lessen the apparent difference, not exaggerate it.

Converging Evidence Confirms Robustness Beyond Scaling Choices

Our findings are robust, as demonstrated by consistent results across diverse model architectures and experimental manipulations. Deep learning models - in which our normalization does not follow the same whole-dataset scaling - exhibit the same inverse relationship between age prediction accuracy and disease detection sensitivity as our linear models. This consistency across different approaches provides compelling evidence that the phenomenon we observe is genuine and not a byproduct of our preprocessing choices.

Furthermore, our systematic manipulation of regularization strength - spanning multiple orders of magnitude - produces clear, coherent patterns that cannot be plausibly attributed to minor variations in scaling. The deliberate experimental adjustments in regularization overwhelmingly dominate any subtle bias from scaling, reinforcing our theoretical framework. In tandem with our feature importance analyses, which demonstrate that increased regularization shifts model focus toward global brain measures (such as total gray matter volume) known to be affected in neurological and psychiatric conditions, the multiple converging lines of evidence collectively validate our findings. This robust

alignment with established neurobiological knowledge and our rigorous matched case-control design further underscore that our observed effects are not driven by methodological artifacts.

Conclusion

In summary, while classical machine learning practice advises fitting scalers on the training data alone, whole-dataset scaling is a common and well-justified approach in large-scale neuroimaging research. Given the enormous sample sizes involved - where imaging-derived features are essentially population constants - and our desire to maintain consistency with parallel studies, any potential bias introduced by this method is quantitatively negligible. Moreover, if such bias were present, it would only serve to enhance the accuracy of the linear models, thereby rendering our central finding - that lower age prediction accuracy can coexist with superior disease detection - more conservative. The consistent and convergent evidence across different model types and experimental conditions strongly confirms that our results are robust and not an artifact of the scaling methodology. Rather than undermining our conclusions, proper contextual understanding of whole-dataset scaling reinforces confidence in the robustness and reliability of our scientific insights.

2. On the Methodology for Bias Correction in Brain Age Gap Analysis

A query was raised regarding our bias correction procedure, which uses parameters derived from the test set (combined patient and control groups). The concern was that this constitutes a form of data leakage, and that correction parameters should be derived from the training set. Our approach, however, is deliberately chosen to optimize for statistical inference within our study sample, which is a distinct goal from optimizing a model for out-of-sample prediction.

Enhancing Statistical Sensitivity by Removing Systematic Noise

The primary purpose of bias correction in brain age studies is to remove the systematic, age-dependent bias (often termed "regression to the mean") that affects virtually all age prediction models, regardless of architecture or training data (Lee and Chen, 2024). This bias - where younger individuals' ages are overestimated and older individuals' ages are underestimated - is a statistical artifact of the prediction process itself, not typically considered a biologically meaningful signal. By applying a correction derived from the specific test sample under analysis (i.e., the combined patient and control groups), we aim to maximally remove this source of systematic noise *from that particular dataset*.

Crucially, for matched patient-control pairs of identical chronological age, applying a linear bias correction ($BAG\_corrected = BAG - (\beta_0 + \beta_1 \cdot Age)$) subtracts the *exact same value* from both the patient and their matched control. This mathematical property ensures that the *raw mean difference* in brain age gap between the patient and control groups remains unchanged by the correction. What *does* change is the pooled standard deviation: by removing the systematic age-related variance, the correction reduces the overall variability within each group, thereby increasing the statistical power (and hence, Cohen's d effect size) to detect the underlying group difference. This explains why brain age studies consistently report enhanced sensitivity after bias correction, even when raw group differences are identical.

Our central aim is a *comparative* analysis of how different models capture disease-related effects. By applying the *same* correction method - derived from the pooled test set - consistently across all models being compared, we ensure a fair comparison. Each model's sensitivity to the biological signal of interest is assessed *after* the common statistical artifact has been maximally removed for the specific sample being analyzed. While absolute effect size values might differ slightly compared to

using a training-set derived correction, the *relative* performance and ranking of the models in detecting disease effects are robustly preserved under this consistent application.

Potential Differential Effects Provide a Conservative Estimate

A valid consideration is whether our test-set-derived bias correction might differentially affect various models. The regression-to-the-mean phenomenon is often more pronounced in simpler linear models compared to more complex, non-linear models. Consequently, applying a correction derived from the test data might slightly improve the apparent accuracy (by reducing age-related prediction errors) of linear models *more* than it improves the accuracy of complex models.

However, this potential differential effect works *against* our central finding. Our key observation is the paradox that models with *lower* age prediction accuracy can yield *larger* disease detection effect sizes. If our bias correction method slightly (and perhaps artificially) *boosted* the accuracy of the linear models relative to the deep learning models, this would only serve to *diminish* the apparent inverse relationship between accuracy and effect size that we report. In other words, our results represent a conservative estimate; employing an alternative correction method (like one derived from the training set) might potentially make the paradoxical gap we observe appear even larger.

Ensuring Demographic Relevance for Accurate Correction

Using correction parameters derived from the test set offers a distinct advantage in terms of demographic alignment. Liang et al. (2019) caution that applying correction parameters derived from a training set can introduce systematic bias if the demographic characteristics (especially age distribution) differ between the training and testing samples - a common situation in neuroimaging research where training data may come from different sources or cohorts.

Our approach sidesteps this potential issue entirely by deriving the correction parameters from the specific pooled sample being analyzed. This ensures perfect demographic alignment between the data used to derive the correction and the data to which it is applied, yielding more accurate individual-level corrections *within our specific study population* and avoiding biases related to demographic mismatch.

Distinguishing From Traditional Data Leakage

It is crucial to differentiate our use of test set information for bias correction from traditional data leakage scenarios in predictive modeling. Standard data leakage occurs when information from the test set inappropriately influences model training or hyperparameter tuning, leading to overly optimistic estimates of generalization performance on truly unseen data. The concern is that the model learns patterns specific to the test set, which won't hold in new applications.

Our context is fundamentally different. We are not using the test set information to *train* the primary age prediction model or to *improve its predictive accuracy* in a way that wouldn't generalize. Instead, we are using the test set solely to estimate and remove a known, systematic *statistical artifact* (age-related bias) *post-hoc* from the predictions generated by already-trained models. The goal is *inferential* - to obtain the most accurate estimate of the underlying group difference (effect size) within our specific study sample by removing confounding variance - not *predictive* in the sense of optimizing out-of-sample performance. Our research question focuses on the *relative* sensitivity of different models to disease effects within this sample. Applying the correction based on the study sample itself gives each model the best chance to reveal its sensitivity to the biological signal by removing the maximal amount of sample-specific statistical noise, thus enabling a fair comparison.

Conclusion

In summary, our bias correction methodology, while differing from standard practice for optimizing out-of-sample prediction, is a statistically sound and methodologically justified approach for our research goal: the comparative analysis of biomarker sensitivity across different models within a matched case-control framework. By deriving the correction from the test set, we effectively reduce systematic statistical noise inherent in age prediction models, thereby enhancing the statistical power to detect group differences. This approach ensures demographic relevance and maintains the integrity of our *comparative* findings across models. Any potential differential impact on model accuracy would render our central conclusion - that lower age prediction accuracy can coexist with superior disease detection sensitivity - more conservative. Therefore, rather than constituting data leakage that invalidates our inferences, our chosen method represents a deliberate strategy to maximize the sensitivity and validity of our comparative effect size estimations within the specific context of this study.

3. On the Performance of Deep Learning Models Trained on Smaller Data Subsets

A query was raised regarding the observation that deep learning models trained on smaller data subsets exhibited large disease-detection effect sizes with seemingly low variance, a finding considered statistically unexpected. This observation, however, is consistent with both our methodological approach and the scientific context.

First, it's important to note that what the query refers to as "small samples" still represents a substantial dataset in deep learning contexts. Even our smallest training portion (25% of 27,000 samples) constitutes approximately 6,750 brain images - a sample size that exceeds many successful deep learning neuroimaging studies. For instance, studies by Aithal and Sinha (2025), Peng et al. (2020), and Cole et al. (2017), have demonstrated robust performance with significantly smaller datasets (1,000-5,000 samples). Modern architectures like ResNet-50 have been shown to learn meaningful representations from datasets of this magnitude, particularly when the signal of interest (like age-related patterns) is relatively strong and distributed.

Clarifying the Visual Representation of Variance

Before addressing the core concern, it's important to clarify a potential misunderstanding about how variance is represented in our figures. The confidence bands shown in our plots are generated using seaborn's linear regression confidence intervals, which represent uncertainty in the regression line itself, not the variance in model performance at each specific training set size. These bands show the 95% confidence interval for the estimated linear relationship between training set size and effect size, rather than directly depicting the variance of individual model runs.

This visualization choice focuses on the trend rather than individual data points, which may have inadvertently contributed to the impression of artificially low variance. The individual data points (which represent separate model runs) do show variability, and upon closer inspection of the figure, the data points for models trained on smaller portions do indeed exhibit slightly greater scatter - consistent with statistical expectations. However, this point-by-point variability is synthesized into the regression confidence bands in our visualization approach.

Statistical Considerations for Effect Size Measurements

The reviewer's concern seems to conflate two different types of variance: variance in model predictive performance and variance in measured effect sizes between patient and control groups. Our primary

metric of interest for the deep learning experiments is not prediction accuracy but rather effect size (Cohen's d) when comparing brain age gaps between patients and controls.

Effect size measurements have different statistical properties than accuracy measurements. They are normalized by the standard deviation of the observed values, which helps stabilize them across different experimental conditions. Additionally, our effect size calculations are performed on relatively large test sets (not the small training sets), which further stabilizes these measurements.

It's also worth noting that the relationship between training set size and effect size shows a consistent negative trend across our experiments. This systematic pattern suggests a genuine phenomenon rather than a methodological artifact. If methodological issues were driving our results, we would expect more erratic patterns without clear directionality.

The Broader Scientific Context

Our findings align with emerging understanding in the field of representation learning for neuroimaging. Recent work has shown that simpler models or models with stronger regularization often outperform highly complex models for certain neuroscientific tasks, particularly when the signal of interest reflects broad, distributed patterns rather than fine-grained local features.

This perspective helps explain why deep learning models trained on small data portions might show larger effect sizes for disease detection: they capture more general, robust brain patterns rather than "over"fitting to fine-grained features specific to chronological age prediction. The consistency in this behavior reflects the fundamental nature of the brain patterns associated with psychiatric and neurological conditions, which often involve widespread structural changes rather than isolated regional effects.

Conclusion: A Valid and Interpretable Pattern

In summary, the "suspiciously low variance" noted by the reviewer can be explained through a proper understanding of our visualization approach, the regularizing properties of modern deep learning architectures, the statistical properties of effect size measurements, and the nature of the brain patterns we're investigating.

Rather than contradicting statistical principles, our findings illuminate the complex relationship between model complexity, training data size, and biomarker efficacy in the specific context of brain age modeling. The consistent patterns we observe across experimental conditions support our central thesis that optimizing models for age prediction accuracy may not yield optimal performance for disease detection - a finding with significant implications for the development of neuroimaging biomarkers.

4. On the Use of SHAP with Correlated Features

A query was raised about the vulnerability of our SHAP-based feature importance analysis to collinearity among neuroimaging features. This is a recognized challenge for some interpretability methods, but our specific implementation was chosen to directly address this issue.

The Context-Specific Nature of SHAP's Limitations

While the reviewer raises an important theoretical concern, it's essential to differentiate between various SHAP implementations and their specific vulnerabilities. The correlation sensitivity issues highlighted in the literature primarily affect KernelSHAP and TreeSHAP implementations, which were

designed for black-box models and tree-based models, respectively. Our analysis employs linear SHAP, which functions differently when applied to linear models like Ridge regression.

Linear SHAP directly uses the model's coefficients to determine feature importance. For a linear model, the SHAP value for a feature is its coefficient multiplied by the feature value minus the mean value. This direct calculation means that linear SHAP doesn't suffer from the same estimation issues that affect other SHAP variants when decomposing non-linear interactions among correlated features.

Ridge regression itself is specifically designed to handle collinearity through L2 regularization, which stabilizes coefficient estimates in the presence of correlated features. When combined with linear SHAP, this provides a more stable interpretation framework than might be suggested by a generalized critique of SHAP methods.

The Different Dynamics of Attribution in Regularized Linear Models

The reviewer's concern would be most applicable if we were using complex non-linear models where interactions between features create ambiguity in attribution. However, our primary interpretability analysis focuses on Ridge regression with varying degrees of regularization. The L2 penalty in Ridge regression explicitly addresses collinearity by shrinking coefficients of correlated features toward each other, effectively handling the very problem the reviewer highlights.

In fact, our analysis leverages this property of Ridge regression to demonstrate how increasing regularization shifts feature importance toward more global brain measures. This shift isn't an artifact of misattribution due to collinearity - it's a direct result of the regularization process itself, which is one of the key mechanistic insights of our paper.

The fact that we observe systematic, interpretable shifts in feature importance as regularization increases (rather than random redistribution of importance) provides evidence that our SHAP analysis is capturing meaningful patterns rather than arbitrary attributions due to collinearity.

The Empirical Support for Our Approach

Our observations align with established neuroanatomical knowledge. As regularization increases, we see greater importance assigned to global brain measures like total gray matter volume, which are widely documented to be affected in numerous neurological and psychiatric conditions. This convergence with prior biological knowledge supports the validity of our feature importance interpretations.

Furthermore, we track these importance shifts across a systematic range of regularization strengths, revealing consistent, gradual transitions rather than erratic jumps that would suggest arbitrary redistribution due to collinearity issues. This systematic behavior supports the robustness of our interpretations.

Appropriately Acknowledged Limitations

While we believe our approach is sound, we acknowledge that no feature attribution method is perfect, especially in high-dimensional, correlated data spaces. However, the central claims of our paper do not rest solely on the precise attribution of importance to specific features. Rather, we use SHAP analysis to demonstrate the general shift from local to global brain measures as regularization increases - a finding that remains valid even with some uncertainty in exact feature attributions.

We have appropriately contextualized our feature importance analysis as providing mechanistic insights into why regularized models might perform better as biomarkers, without making strong causal claims about specific brain regions. This measured approach to interpretation acknowledges the inherent limitations of any feature attribution method.

Conclusion

In summary, while the reviewer raises a general concern about SHAP analysis with correlated features, this concern is less applicable to our specific implementation using linear SHAP with Ridge regression. The regularization process itself addresses collinearity, and our systematic analysis across regularization strengths reveals consistent, interpretable patterns that align with neurobiological knowledge.

Rather than dismissing the limitations of feature attribution methods, we have conducted our analysis in a way that mitigates these concerns through appropriate model selection and interpretation. The insights gained from this analysis contribute valuable understanding to why simpler, more regularized models might provide better biomarkers for brain disorders, without overstepping the bounds of what can be reliably interpreted from our data.

This nuanced approach to model interpretation represents a strength of our analysis, not a limitation, and provides a solid foundation for the mechanistic explanations offered in our manuscript.

5. On the Relationship Between Model Fit and Biomarker Utility

A query was raised suggesting a misunderstanding of overfitting and underfitting, based on our finding that models with better validation performance (for age prediction) can be poorer biomarkers. This critique stems from a conventional machine learning framework that does not account for the specific context of our study, where the optimization task is a proxy for the ultimate scientific goal.

Distinguishing Between Different Optimization Objectives

In standard machine learning applications, the training and validation objectives are identical – we train a model to predict Y from X, and validate it on its ability to do exactly that task. In our case, however, we're examining a more complex scenario where the ultimate goal (disease detection) differs from the proxy task (age prediction). This creates a situation where improving performance on the proxy task (as measured by validation error) can actually reduce performance on the ultimate task.

This isn't a confusion about overfitting or underfitting – it's a sophisticated understanding of the limitations of proxy tasks. The deep learning models in our study are performing exactly as expected on their assigned task (age prediction), showing decreasing validation error with more data and training. There's no misunderstanding here. Instead, we're revealing that optimizing for this proxy task may not yield optimal performance for the clinical application of detecting disease.

The Special Case of Brain Age Models

Brain age modeling presents a unique scenario where the standard overfitting/underfitting framework requires additional nuance. Unlike typical supervised learning problems where we directly optimize for the outcome of interest, brain age models involve a two-step process: (1) train models to predict chronological age in healthy individuals, then (2) measure deviations from predicted age in clinical populations as a biomarker.

This indirect approach means that the relationship between model complexity, regularization, and biomarker efficacy doesn't follow conventional machine learning intuitions. A model that fits age prediction extremely well might be capturing fine-grained, age-specific features that are less relevant to disease processes, while a more constrained model might focus on broader structural patterns more sensitive to pathological changes.

The Empirical Evidence

Our empirical findings provide compelling evidence for this perspective. We consistently observe that as models become more accurate at age prediction (through increased data, longer training, or less regularization), they often become less sensitive to disease-related brain differences. This observation holds across different model architectures and experimental conditions, suggesting a fundamental trade-off rather than a methodological error.

Our feature importance analysis further supports this interpretation, showing that highly regularized models prioritize global brain measures like total gray matter volume, which are known to be affected in many neurological and psychiatric conditions. In contrast, models optimized for age prediction focus on more specific regional features that may be excellent age predictors but less relevant to disease processes.

Regularization vs. Undertraining: A Critical Distinction

Our approach differs substantively from the "loose fitting" hypothesis criticized by Hahn (2021). We're not advocating for undertraining models, which could indeed lead to arbitrarily poor generalization. Instead, we're systematically applying regularization to fully trained models, constraining their capacity to combine age-relevant features in complex ways.

This distinction is crucial because regularized models still learn meaningful patterns relevant to the target variable, but with deliberate constraints on complexity. The relationship we observe between regularization strength and biomarker efficacy is systematic and interpretable, not random or arbitrary as would be expected from undertraining.

Conclusion

What the reviewer interprets as confusion about overfitting is actually a sophisticated understanding of the difference between statistical optimization and clinical utility. In clinical applications, the "best" model isn't necessarily the one with lowest prediction error on the proxy task, but rather the one that maximizes sensitivity to clinically relevant variations.

Our findings don't contradict machine learning principles – they extend them by highlighting how the standard validation paradigm must be adapted when the ultimate goal differs from the training objective. This insight has profound implications for developing clinically useful biomarkers, suggesting that we should sometimes deliberately constrain models to enhance their sensitivity to disease-relevant features, even at the cost of reduced accuracy on the proxy task.

This perspective represents an advancement in the application of machine learning to clinical problems, not a misunderstanding of basic concepts. The reviewer's critique demonstrates precisely the conventional thinking our work aims to move beyond – that maximizing validation performance on a proxy task automatically optimizes a model for all potential applications.

Supplementary Note B: Empirical Validation of Key Methodological Choices

To address methodological concerns, we conducted systematic robustness analyses across diagnostic conditions. For each condition, we created matched patient-control pairs based on demographics. Brain-age models were trained on healthy participants only (n=5,000 training, n=1,000 validation), then applied to matched pairs to calculate brain-age gaps. We used Ridge regression with systematically varied regularization (α=1e+02, 1e+04).

1. Scaling Approach Robustness: We compared two standardization approaches: (a) training-only scaling (StandardScaler fitted on training set, applied to all datasets), and (b) whole-dataset scaling (StandardScaler fitted on combined training+test data). For each approach, we trained Ridge models, calculated brain-age gaps, and measured disease detection effect sizes (Cohen's d). We quantified robustness using relative differences: |Effect_A - Effect_B|/Effect_A × 100%.

   Results: Relative differences in effect sizes were consistently negligible (< 0.1%), with mean differences ranging from 0.001% to 0.089% across conditions. R^2 differences were similarly minimal (< 0.1%).

2. Bias Correction Method Robustness: We tested four bias correction approaches: (a) no correction (raw brain-age gaps), (b) training-derived (linear regression fitted on training set: bias = $\beta_0 + \beta_1 \times$age), (c) test-derived (linear regression fitted on healthy test set), and (d) controls-derived (linear regression fitted on matched controls). Corrected gaps were calculated as: BAG_corrected = BAG_raw - predicted_bias.

   Results: Differences in effect size increase with over-regularisation remained below 6% across all methods. Cross-method effect size correlations exceeded 0.99, indicating high consistency. The brain-age paradox (higher regularization → lower R^2 but larger effect sizes) persisted across all correction methods.

3. SHAP Collinearity Mitigation: We validated Monte-Carlo (MC) resampling (effectively bootstrap as n<<N) for feature importance analysis using Linear SHAP on Ridge regression models. First, we quantified collinearity by calculating pairwise correlations among 1,440 brain features. Then we compared single SHAP runs (n=10, different random seeds) versus bootstrap averaging (n=10 iterations, 1,000 samples each). We measured stability using: (a) top-10 feature overlap (Jaccard index), (b) rank correlation (Spearman's ρ), and (c) coefficient of variation.

   Results: Despite high collinearity (6,000+ feature pairs with $|r| > 0.7$, representing 0.64-0.66% of all pairs), MC resampling achieved robust stability. Single runs showed high instability (mean CV = 0.065-0.070), while MC resampling achieved 68-78% top-10 feature overlap and rank correlations of 0.73-0.75.
